# Periareolar approach in video-assisted thoracoscopic surgery for right middle lobectomy: a novel technique

**DOI:** 10.1007/s00464-024-11121-1

**Published:** 2024-08-05

**Authors:** Zhangfan Mao, Ping Dong, Qing Zhou, Shaowen Zhang

**Affiliations:** 1 Department of Thoracic Surgery, Renmin Hospital of Wuhan University, 238 Jiefang Road, Wuhan, 430060 People’s Republic of China; 2 Department of Neurology, Tongji Hospital Affiliated to Tongji Medical College, Huazhong University of Science and Technology, 1095 Jiefang Avenue, Wuhan, 430030 People’s Republic of China

**Keywords:** Periareolar incision, Video-assisted thoracoscopic surgery, Transareolar port, Uniport, Periareolar port

## Abstract

**Background:**

Uniportal thoracoscopic right middle lobectomy (RML) poses greater technical challenges than other lobectomies. Although two-port thoracoscopy offers convenience, it results in heightened surgical trauma and scarring. The periareolar incision is rarely used in lobectomy while known for its cosmetic advantages. This study presents the periareolar access (combining a periareolar port and a 1-cm port) for video-assisted thoracoscopic surgery (VATS) in RML, comparing it with the traditional uniportal technique in both male and female patients.

**Methods:**

Eighty patients who underwent RML were randomly divided into two groups: the periareolar VATS (PV) approach (*n* = 40) and the uniportal VATS (UV) approach (*n* = 40) from August 2020 to February 2023. All patients were followed up for 1 year and clinical data were collected and analyzed.

**Results:**

No significant differences in complications, blood loss, duration of chest tube placement, and length of postoperative hospital stay were observed between two methods. However, the PV group exhibited significantly shorter operative time, reduced postoperative visible scarring and lower visual analogue scores (VAS) for postoperative pain (*P* < 0.05). Additionally, the PV group demonstrated significantly higher cosmetic and satisfaction scores at the 6-month postoperative assessment (*P* < 0.05). Notably, breast ultrasound follow-up revealed two cases injuries of the mammary glands in female patients, and sensory function of most nipple and areola remained intact except two cases in all PV group patients.

**Conclusions:**

Periareolar VATS emerges as a promising alternative approach for RML, providing clear benefits in pain management and cosmetic outcomes, while maintaining safety and convenience.

**Graphical Abstract:**

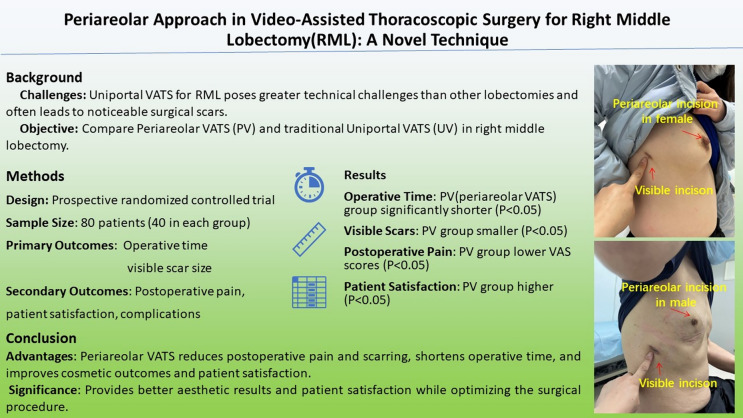

**Supplementary Information:**

The online version contains supplementary material available at 10.1007/s00464-024-11121-1.

The advancement of video-assisted thoracoscopic surgery (VATS) has driven the evolution of thoracoscopic lobectomy techniques, progressing from three ports to two ports, uniport, and even the "super uniport" with a 2-cm incision [1, 2]. However, the adoption of smaller ports has increased surgical complexity and extended operative durations, especially in right middle lobectomy (RML). Uniportal thoracoscopic RML is more challenging than other uniportal thoracoscopic lobectomies due to limited distance between the incision and the hilum, restricting instrument maneuverability and endo-stapler navigation. Moreover, the straight orientation of the endo-stapler poses challenges in accessing hilar structures, elevating the risk of major injuries. To address these difficulties, larger uniportal incisions are sometimes required to dissect and staple veins, bronchi, arteries, and fissures.

In contrast, the two-port VATS approach enhances instrument flexibility, reducing surgical complexity and risks compared to uniportal procedures. The periareolar incision, known for its cosmetic benefits and pain relief postoperatively, has been extensively employed in endoscopic thyroidectomy surgery and minimally invasive cardiac Surgery [3, 4]. Although periareolar VATS has been applied in males for thymectomy, sympathectomy, and simple lung resection, its use in lobectomy cases has been limited [5–7]. Particularly for female patients, there is a lack of reports on periareolar VATS.

In this study, we introduced the periareolar incision as an access point for VATS, accompanied by an additional 1-cm port to facilitate the RML procedure. The aim of this study was to assess whether this innovation approach may improve the RML process.

## Material and methods

### Study design

This randomize, single blind, two arm clinical trial was conducted in the Department of Thoracic Surgery at Renmin Hospital of Wuhan University, spanning from August 2020 to February 2023 in the same surgery team. After the baseline assessment, an independent investigator randomized eligible patients in a 1:1 ratio in each group.

The Primary outcome of our study are the reduction in operative time and the size of the visible surgical scar. We hypothesize that the periareolar VATS (PV) approach would result in a significantly shorter operative time and smaller visible scar compared to the traditional uniportal VATS (UV) approach. The secondary outcomes include:

- Postoperative pain (assessed using Visual Analog Scale (VAS) scores)
- Patient satisfaction (evaluated through satisfaction scores)
- Complications such as blood loss, duration of chest tube placement, and length of postoperative hospital stay

These outcomes measures were selected to comprehensively assess the clinical benefits and patient-centric advantages of the periareolar VATS approach.

Based on our team’s clinical experience and data, as well as previous studies on uniportal thoracoscopic right middle lobectomy (RML) [1, 9], we anticipated a 15-min difference in operative time with an expected standard deviation of 20 min between the periareolar VATS (PV) and uniportal VATS (UV) approaches. We used PROC POWER in SAS to calculate that a sample of 72 evaluable patients (36 in periareolar VATS, 36 in uniportal VATS) would be needed to provide 80% power at a significance level of 5%. To ensure robustness and account for potential dropouts, we decided on a sample size of 40 patients per group, totaling 80 patients.

Patients were randomly divided into two groups: periareolar VATS (PV) approach (*n* = 40) and conventional 3-cm uniportal VATS (UV) procedure (*n* = 40).

#### Inclusion criteria

Patients aged 18–75 years, presenting with pulmonary lesions of less than 1 cm in diameter, and no significant swelling of lymph nodes in the chest were observed.

#### Exclusion criteria

Patients with severe comorbidities, previous thoracic surgery, or those requiring conversion to open surgery.

The study adhered to ethical guidelines, including the World Medical Association Declaration of Helsinki and the Declaration of Istanbul, and received approval from the ethics committee of Renmin Hospital of Wuhan University (2020-1-X-67). All participating patients provides informed consent. Our study adhered to CONSORT 2010 statement: updated guidelines for reporting parallel group randomize trial [8].

### Patients and surgical procedure

Before surgery, all patients underwent routine preoperative tests, revealing no contraindications for the surgical procedures. Surgeries were performed under combined intravenous and inhalation anesthesia, utilizing double-lumen tracheal intubation and selective one-lung ventilation. Patients were positioned in the lateral decubitus position for surgery. The perioperative pain management protocol was the same for both groups [1, 2].

### Periareolar thoracoscopic port

**Fig. 1:**
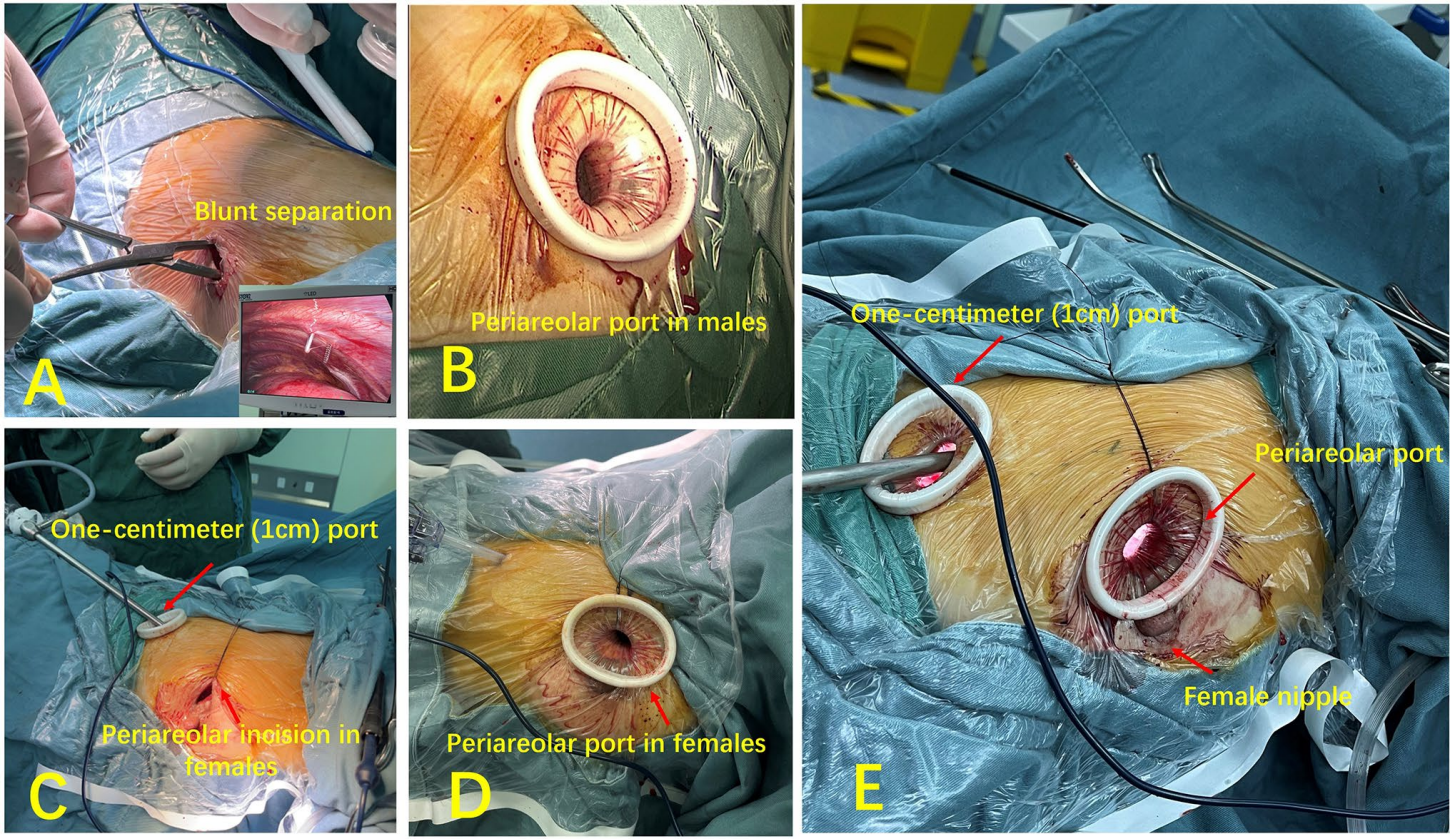
Periareolar thoracoscopic port for both male and female patients. **A** The male areolar incision was made by blunt separation and the periareolar port on the chest wall was more medial than the traditionally used procedure. **B** In the female patients, we bluntly separated the subcutaneous fat tunnel between the areolar incision and the proposed intercostal incision. **C** After placing a wound protector, we formed a periareolar surgical port of approximately 2 cm × 1.5 cm to 2.5 × 2 cm. This photograph is of the male. **D** In the female patients, the periareolar port will pass through the subcutaneous tunnel at some angles to the chest wall in a way different from that of the male. **E** The periareolar port combined with a 1-cm port to form the two-port VATS model. The surgical instruments could be flexibly applied in the two ports. *VAST* Video-assisted thoracoscopic surgery

For both male and female patients, the periareolar incision was created as an approximately 2-cm arc-shaped cut, covering around one-third of the lateral border of the areola (Fig. 1A and B). To minimize bleeding, a subcutaneous injection of 1% adrenaline solution was administered at the areolar incision site, followed by a subcutaneous intercostal muscle and nerve block using 1% ropivacaine. In the case of male patients, the chest wall and intercostal muscles were bluntly separated, allowing access into the thoracic cavity at the fourth intercostal space (Fig. 1A). Subsequently, the intercostal incision was made, and a wound protector (HK-60/70, Beijing Hangtian Kadi Technology Development Institute) was inserted to create an approximately 2 cm × 1.5 cm surgical port (Fig. 1C).

In female patients, a subcutaneous tunnel was meticulously created to protect breast tissue, connecting the areolar incision to the intended intercostal incision site within the thoracic cavity (Fig. 1B, D, E). This port exhibited a different angle in relation to the chest wall compared to the periareolar port used in male patients and the conventional uniport (Fig. 1D). The procedure for creating the female periareolar thoracoscopic port was demonstrated in Video 1.

### Periareolar two-port VATS for right middle lobectomy

To create the 1-cm port, a gentle 1-cm incision was made at the seventh intercostal space along the midaxillary line, commonly known as a "super port" [1]. After introducing a wound protector, a port measuring 2 cm × 1.5 cm was established. This port, combined with the periareolar port, formed a two-port VATS model (Fig. 1E). This configuration allowed flexible use of surgical instruments between the two ports. During dissection, a thoracoscopic lens and double-joint clamp were placed in the 1-cm port, while an electrocautery hook and aspirator were positioned in the periareolar port. The endo-stapler could be used in either port, with other instruments adjusted accordingly, enabling a more flexible and appropriately angled operation. In female patients, due to variations in breast shape and intercostal width, the final size and angle of the areolar port differed, allowing surgeons to make flexible adjustments in the application of instruments between the two ports.

The procedure initiated with the lateral and posterior retraction of the right middle lobe to expose and dissect the middle pulmonary vein. Following vein dissection, it was divided using the endo-stapler through one of the two ports. If the endo-stapler was employed in the 1-cm super port, the thoracoscope was shifted to the periareolar port. The middle lobe bronchus, located posteriorly and surrounded by lymph nodes, was dissected by removing lymph nodes with an electrocautery hook or harmonic scalpel, followed by bronchus division using the endo-stapler. Once the bronchus was divided, branches of the pulmonary arteries within the horizontal fissure were exposed and managed by either clipping with hem-o-locks, division with an endo-stapler, or transection together with the horizontal fissure using the endo-stapler. The anterior oblique fissure was dissected using an electric hook, harmonic scalpel, or division with an endo-stapler after creating a tunnel. If the pulmonary fissure was well developed, arteries were treated preferentially. A demonstration of the RML procedure can be found in Video 2.

**Fig. 2:**
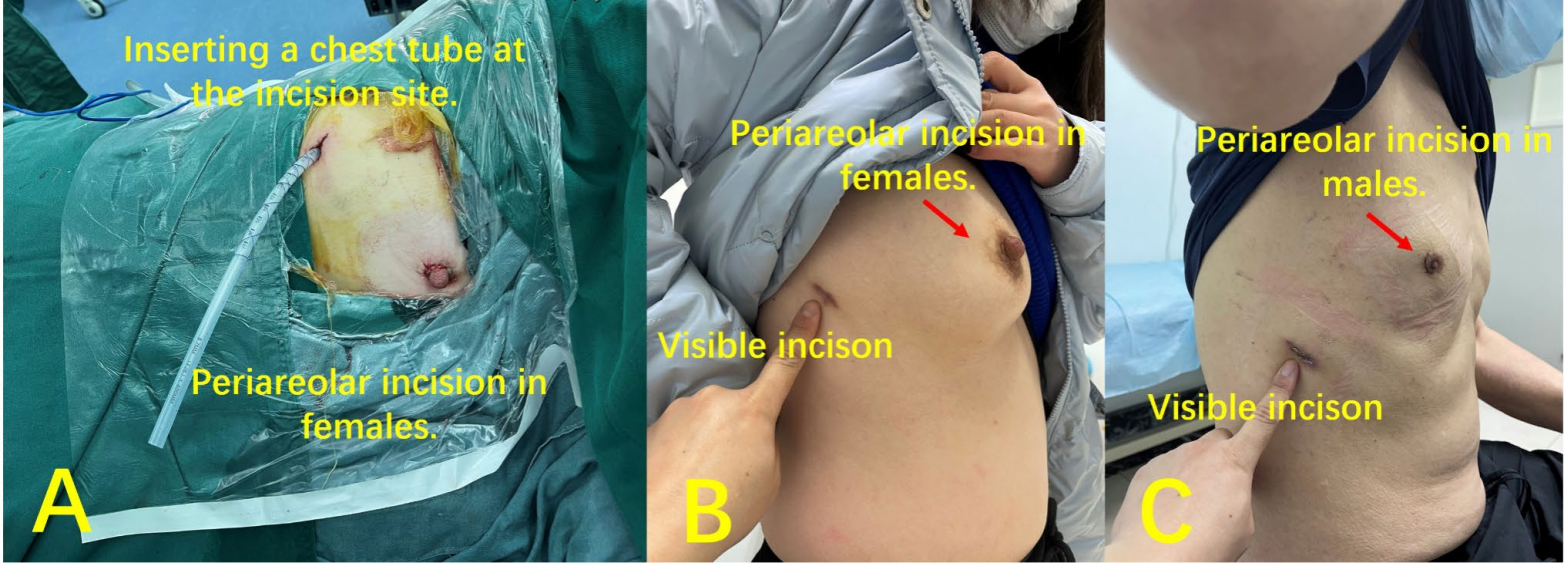
The periareolar incision for patients with malignant lesions. **A** A 16-F chest tube was ultimately placed at the 1-cm port. The respective scars of the two ports in females (**B**) and males (**C**). The periareolar incision shows a scarless effect and another port shows a smaller visible scar

For patients with malignant lesions, hilar and mediastinal lymph node dissection was performed, and air-leak testing was conducted. The right middle lobe was placed into a plastic bag and removed through either the periareolar port or 1-cm port. If the lobe was large, it was divided into two pieces using the endo-stapler. Finally, a 20-F chest tube was inserted through the 1-cm port (Fig. 2A).

The usual uniportal incision is approximately 3 cm in length and uniportal RML has conventionally been employed in the UV group. The same 20-F chest tube was inserted through the uniport after surgery.

### Data collection and follow-up

During the 12-month postoperative follow-up period, all patients were closely monitored and actively participated in the data collection process. The whole process was supervised by independent research assistants. A detailed questionnaire was completed by each patient, collecting valuable information regarding their postoperative experience, satisfaction, and cosmetic outcomes. Specifically, the collected data encompassed the following aspects:

- Cosmetic Score (Verbal Response Scale, VRS): 1: Dissatisfied/2: Accepted/3: Satisfied/4: Perfect;
- Postoperative Pain Score (Visual Analog Scale, VAS): ranging from 0 (indicating no pain) to 10 (representing the worst pain imaginable);
- Satisfaction Score: Very Satisfied (9–10); Satisfied (6–8); Dissatisfied (3–5); Very Dissatisfied (0–2).

Additionally, the potential recurrence of tumors was rigorously evaluated through chest computed tomography (CT) scans. Patients were recommended to undergo regular chest CT scans every 3 months, coupled with comprehensive general examinations every 6 months during the follow-up period. Notably, individuals in the periareolar VATS group received an additional breast B-ultrasound examination every 3 months as part of their tailored follow-up protocol. This comprehensive approach facilitated the systematic monitoring and assessment of patient outcomes and potential recurrence.

### Statistical analysis

All statistical analyses were conducted using SPSS 22.0 statistical software (IBM Corporation, Armonk, NY, USA) by an independent statistician. The D’Agostino-Pearson test was used to evaluate whether the data conforms to a normal distribution. Comparisons between two groups were performed with Student’s *t*-test or Mann–Whitney *U*-test. Pearson chi-square test and Fisher’s exact test were used for categorical variables comparisons. Data were presented as mean ± standard deviation (SD) or median (range) or number (percentage). A *P*-value of less than 0.05 was considered statistically significant.

## Results

All 80 patients underwent successful thoracoscopic RML, comprising 40 cases of uniportal thoracoscopic RML and 40 cases of periareolar two-port thoracoscopic RML. Notably, 16 RML procedures were performed through the female areola. Clinical characteristics for both groups are presented in Table 1. No severe complications, perioperative mortality, or conversions to thoracotomy were noted in either group. Additionally, there were no significant differences in blood loss, chest tube indwelling time, or postoperative hospital stay between the two groups (Table 2). Notably, the operative time was significantly shorter in the periareolar two-port VATS (PV) group compared to the uniportal VATS (UV) group (66 ± 25 min vs. 81 ± 22 min, *P* < 0.05) (Table 2). While two cases in the UV group required conversion to two-port VATS, no additional ports were needed in the PV group. In addition, we found that the procedure with the periareolar incision in females is slightly more challenging and takes a bit longer time compared to males. The differences in total operating time were not statistically significant (76 ± 27 min vs. 71 ± 21 min, *P* > 0.05).

**Table 1:** Clinical characteristics of both PV and UV groups at baseline

Variables	PV group (*n* = 40)	UV group (*n* = 40)	*P* value
Age (years), mean ± SD	44.60 ± 14.37	43.30 ± 13.88	> 0.05
Female, *n* (%)	16 (40.00)	13 (32.50)	> 0.05
BMI, mean ± SD	22.77 ± 2.94	23.82 ± 3.53	> 0.05
Smoking history, *n* (%)	10 (25.00)	5 (12.50)	> 0.05
Drinking history, *n* (%)	6 (15.00)	8 (20.00)	> 0.05
Sedatives using, *n* (%)	2 (5.00)	2 (5.00)	> 0.05
Diabetes history, *n* (%)	7 (17.50)	6(15.00)	> 0.05

Statistic for age and BMI: Student’s *t*-test

Statistic for other variables: Pearson chi-square test and Fisher’s exact test

*PV* Periareolar two-port video-assisted thoracoscopic surgery (VATS), *UV* uniportal VATS, *BMI* body mass index, *SD* standard deviation

**Table 2:** Comparisons of postoperative outcomes between PV and UV groups

Variables	PV group (*n* = 40)	UV group (*n* = 40)	*P* value
Operative time (min), mean ± SD	66 ± 25	81 ± 22	< 0.05
Changing approaches in operation, *n* (%)	0 (0.00)	2 (5.00)	> 0.05
Blood loss (mL), mean ± SD	68 ± 31	84 ± 77	> 0.05
Duration of postoperative drainage (days), mean ± SD	4.9 ± 1.8	4.3 ± 1.9	> 0.05
Postoperative hospital staying (days), mean ± SD	7.6 ± 2.6	9.1 ± 2.4	> 0.05
Complications, *n* (%)			
arrhythmia	3 (7.50)	4 (10.00)	> 0.05
Pleural effusion	4 (10.00)	2 (5.00)	> 0.05
Pneumonia	2 (5.00)	3 (7.50)	> 0.05
Air leaks	1 (2.50)	2 (5.00)	> 0.05
Atelectasis	0 (0.00)	1 (2.50)	> 0.05
Incision delayed healing	2 (5.00)	3 (7.50)	> 0.05
Size of the visible scar (cm), mean ± SD	1.6 ± 0.4	4.0 ± 0.7	< 0.05
VAS at 1st week (0–10), mean ± SD	1.8 ± 1.0	2.5 ± 1.1	< 0.05
VAS at 1st month (0–10), mean ± SD	1.2 ± 0.6	2.1 ± 1.0	< 0.05
Satisfaction score, mean ± SD	9.1 ± 0.9	7.2 ± 1.1	< 0.05
Cosmetic score, mean ± SD	3.6 ± 0.6	2.9 ± 0.7	< 0.05
Incision numbness at 1st month, *n* (%)	2 (5.00)	9 (22.50)	< 0.05
Pathological diagnosis, *n* (%)			
Benign lesions	3 (7.50)	4 (10.00)	
Adenocarcinoma	34 (85.00)	33 (82.50)	
Squamous cell carcinoma	3 (7.50)	3 (7.50)	
TNM stage in malignant lesions, *n* (%)			
T1N0M0	36 (90.00)	34 (85.00)	
T1N1M0	1 (2.50)	1 (2.50)	
T1N2M0	0 (0.00)	1 (2.50)	
Number of dissected lymph nodes, median (range)	9 (6–16)	11 (8–17)	> 0.05
Recurrence, *n* (%)	0 (0.00)	0 (0.00)	

Statistic for operative time, blood loss, duration of postoperative drainage, postoperative hospital staying, size of the visible scar, VAS at 1st week, VAS at 1st month, satisfaction score, cosmetic score and number of dissected lymph nodes: Student’s *t*-test

Statistic for other variables: Pearson chi-square test and Fisher’s exact test

*PV* Periareolar two-port video-assisted thoracoscopic surgery (VATS), *UV* uniportal VATS, *VAS* visual analogue scores for postoperative pain (range: 0–10), *SD* standard deviation

All periareolar incisions exhibited inconspicuous surgical scars (Fig. 2B and C). Besides, the PV group exhibited significantly lower levels of visible scarring, incision numbness, and VAS for postoperative pain at one week and one month in comparison to the UV group (Table 2). Two cases with delayed healing in the areolar incision were observed, one in a male patient and one in a female patient. In female patients, two cases breast gland injury was detected by B-ultrasound after surgery and spontaneously healing, and there were two cases abnormal sensory function in the PV group, and no discernible alterations in the shape of nipple or areola. Ultimately, at the one-year follow-up, the cosmetic score and satisfaction score in the PV group were significantly higher than those in the UV group.

Concerning postoperative pathology, the PV group encompassed 3 benign and 37 malignant lesions, whereas the UV group had 4 benign and 36 malignant lesions (Table 2). Among the malignant lesions, 67 were adenocarcinomas, 6 were squamous cell carcinomas, and 70 were classified as stage I (T1N0M0), 2 as stage II (T1N1M0), and 1 as stage III (T1N2M0). There were no significant differences in the number of dissected lymph nodes between the two groups.

## Discussion

Uniportal VATS is a widely used minimally invasive technique, yet it often leads to noticeable surgical scars. Uniportal thoracoscopic RML poses particular challenges due to restricted instrument maneuverability and visualization. Various approaches have been attempted to optimize RML, such as adopting larger uniportal incisions at different intercostal spaces [9]. However, these larger incisions raise cosmetic concerns.

Periareolar incisions confer significant cosmetic benefits, utilizing the thin, flexible, and pigmented nature of areolar skin to effectively conceal postoperative scars. Its limited elasticity yields smooth incisions after healing, resulting in periareolar thoracoscopic ports being virtually scarless [5, 10]. In this study, most periareolar incisions exhibited no visible surgical scars post-surgery, with patients reporting low VAS scores for postoperative pain. This may be attributed to reduced damage to intercostal nerves during blunt separation and smaller incision, in contrast to traditional uniportal incisions. The periareolar port acts as an additional surgical access point, enabling versatile application of instruments and endo-staplers from diverse angles. This flexibility proves especially advantageous in cases involving thoracic adhesions, hypoplastic lung fissures, and bleeding control, effectively overcoming the limitations of uniportal VATS in RML. The operative time was significantly shorter in the PV group in this study.

The extraction of lung lobes after surgery may be of concern from small port. In our experience, the middle lobe is relatively small and does not typically require division into two parts. We encountered only one case where the lobe was divided, and in that instance, we avoided the tumor area and cut the normal lung tissue to make the middle lobe smaller for extraction. Therefore, this procedure does not affect the pathology result or tumor staging.

To address concerns about mammary gland damage and inflammation in female patients, a subcutaneous tunnel was meticulously created from the areolar incision to the intercostal incision leading to the thoracic cavity. This technique effectively prevented breast damage, as confirmed by postoperative B-ultrasound follow-up. Although some patients exhibited post-surgery breast indurations, these generally resolved within a month. Poor healing of the areolar incisions was rarely observed in either male or female patients. Consistent with previous studies, this study demonstrates that the periareolar two-port approach not only yields less visible scarring [7] and lower postoperative pain levels compared to uniportal VATS [11] but also reduces operative time. Therefore, the periareolar port not only enhances cosmetic outcomes and minimizes pain but also optimizes the RML surgical procedure.

We acknowledge the initial exploratory nature of this study and its limitations. This preliminary phase was designed to evaluate the feasibility and safety of the periareolar VATS approach. The findings from this study will inform future larger-scale, multi-centered studies. These future studies will be registered in international clinical trial registries to ensure comprehensive evaluation and broader applicability.

## Conclusions

In summary, the periareolar incision emerges as a promising choice for thoracoscopic operative access, distinguished by benefits in pain management and cosmetic outcomes. Moreover, the application of periareolar two-port VATS in RML could optimize the surgical procedure while maintaining safety and convenience.

### Supplementary Information

Below is the link to the electronic supplementary material.

Supplementary file1 (MP4 11261 KB)

Supplementary file2 (MP4 40657 KB)
